# Autologous Fat Grafting Versus Alloplastic Materials in Post-Traumatic Urogenital Reconstruction

**DOI:** 10.7759/cureus.94992

**Published:** 2025-10-20

**Authors:** Fatima Gilani, Muhammad Rizwan, Ikram Haq, Zeeshan Umar, Muhammad Moazzam Farooq, Umar Aziz, Hira Khan, Wajahat Ullah khan

**Affiliations:** 1 Emergency Medicine, Shifa International Hospital, Islamabad, PAK; 2 Medicine and Surgery, Ayub Teaching Hospital, Abbottabad, PAK; 3 Neurological Surgery, Lady Reading Hospital Medical Teaching Institution (MTI), Peshawar, PAK; 4 Trauma and Orthopaedics, Glangwili General Hospital, Carmarthen, GBR; 5 Surgery, Khyber Teaching Hospital, Peshawar, PAK; 6 Emergency Medicine, Pak Emirates Military Hospital (PEMH), Rawalpindi, PAK; 7 Plastic and Burn Unit, Hayatabad Medical Complex Peshawar, Peshawar, PAK; 8 Trauma and Orthopaedics, Services Hospital Lahore, Lahore, PAK; 9 Forensic Medicine, Women Medical College, Abbottabad, PAK; 10 General Surgery, Hayatabad Medical Complex Peshawar, Peshawar, PAK

**Keywords:** aesthetic results, alloplastic materials, autologous fat grafting, patient satisfaction, reconstructive outcomes, urogenital trauma

## Abstract

Background: Traumatic soft tissue injuries to the urogenital area need reconstructive procedures that minimize complications while restoring both shape and function.

Objective: To compare the reconstructive outcomes of autologous fat grafting versus alloplastic materials in the repair of post-traumatic urogenital soft tissue defects with respect to complication rates, aesthetic outcomes, and patient-reported satisfaction in a collaborative plastic surgery-urology clinical setting.

Methodology: This was a descriptive, multicenter observational study conducted from November 2022 to October 2024 at Shifa International Hospitals, Islamabad, along with Khyber Teaching Hospital, Peshawar, and Hayatabad Medical Complex, Peshawar. Out of 204 patients with post-traumatic urogenital abnormalities who were at least 18 years old, 106 had autologous fat grafting, and 98 had alloplastic repair. At six and 12 months, prospective data were gathered on demographics, injury features, surgical parameters, postoperative complications, cosmetic results (as judged by a panel of blinded people), and patient satisfaction (5-point Likert scale). IBM Corp. Released 2020. IBM SPSS Statistics for Windows, Version 26. Armonk, NY: IBM Corp. was used for statistical analysis, which includes chi-square, t-tests, and multivariable regression.

Results: Mean surgical time was shorter in the fat grafting group (92.4 ± 18.1 min vs. 103.7 ± 20.9 min, p=0.002), with reduced hospital stay (1.8 ± 0.6 vs. 2.4 ± 1.1 days, p=0.001). Complication rates were significantly lower for fat grafting: infection in 5/106 patients (4.72%) vs. 14/98 (14.29%), p=0.018; extrusion in 0/106 (0%) vs. 9/98 (9.18%), p=0.003; fibrosis in 4/106 (3.77%) vs. 12/98 (12.24%), p=0.021. Excellent aesthetic scores at 12 months were achieved in 61/106 patients (57.55%) vs. 31/98 (31.63%), p=0.001, while very high satisfaction was reported by 58/106 (54.72%) vs. 29/98 (29.59%), p=0.001.

Conclusion: Autologous fat grafting yields superior functional and aesthetic outcomes with fewer complications compared to alloplastic materials in post-traumatic urogenital reconstruction.

## Introduction

Traumatic blunt, penetrating, or iatrogenic soft tissue injuries of the urogenital area provide major reconstructive problems [1,2]. In addition to affecting sexual and urinary function, these injuries have significant psychological and cosmetic repercussions for the patients [3]. The urogenital region's unique anatomy and functional complexity need customized surgical techniques that minimize long-term morbidity while restoring both form and function [4]. 

Alloplastic materials, such as silicone implants, acellular dermal matrices, and synthetic meshes, have long been used in reconstructive techniques for these kinds of lesions [5]. Despite being useful for structural repair, these materials have risks, especially in regions with impaired vascularity or prior contamination, including infection, extrusion, foreign body response, and fibrosis [6]. On the other hand, autologous fat grafting has become a viable substitute for soft tissue repair in a variety of anatomical locations [7,8]. 

Fat grafting, which uses the patient's own adipose tissue, has the advantages of biocompatibility, flexibility, and possible regenerative benefits while being less immunogenic [9]. Graft survival has increased, and its use in reconstructive surgery has broadened due to recent developments in fat harvesting, processing, and injection procedures [10,11]. With lower complication rates than synthetic implants, autologous fat grafting especially provides the possibility for shape restoration, scar remodeling, and enhanced tissue pliability in urogenital repair. However, there is a dearth of extensive, comparable data, and its significance in treating post-traumatic urogenital abnormalities is still little understood [12].

Selecting the best reconstructive technique is essential because of the urogenital region's functional significance and anatomical sensitivity. Planning and carrying out treatments are improved by a multidisciplinary approach that includes urologists and plastic surgeons, especially when striking a balance between functional and cosmetic objectives. Despite increased interest, there is currently little data that directly compares autologous fat grafting with alloplastic restoration in post-traumatic urogenital soft tissue repair, particularly in healthcare settings with restricted resources. The objective of this study was to compare the reconstructive outcomes of autologous fat grafting versus alloplastic materials in the repair of post-traumatic urogenital soft tissue defects with respect to complication rates, aesthetic outcomes, and patient-reported satisfaction in a collaborative plastic surgery-urology clinical setting.

## Materials and methods

Study design and setting

This descriptive, observational multicenter study was conducted over two years (November 2022 to October 2024) at Shifa International Hospitals, Islamabad, Khyber Teaching Hospital, and Hayatabad Medical Complex, Peshawar. This study was designed to descriptively compare clinical outcomes and complication rates between autologous fat grafting and alloplastic reconstruction for post-traumatic urogenital soft tissue defects. A multidisciplinary team of plastic surgeons and urologists collaboratively evaluated and managed all cases to ensure uniform decision-making and surgical techniques across sites.

Inclusion and exclusion criteria

Patients with post-traumatic urogenital soft tissue anomalies requiring surgical repair were included, regardless of gender, and they had to be at least 18 years old. Eligibility was limited to patients receiving alloplastic material-based reconstruction or autologous fat grafting. Patients with systemic infections, congenital urogenital abnormalities, active cancer in the area, or unfinished follow-up data for at least a year after surgery were excluded.

Sample size

A total of 204 patients were enrolled through convenience sampling from eligible inpatient and outpatient populations across the three tertiary care centers, Departments of Plastic Surgery and Urology at Shifa International Hospitals, Khyber Teaching Hospital, and Hayatabad Medical Complex. The multicenter observational design and the intent to include all consecutive patients who met the inclusion criteria over the two-year study period justified the use of this sampling approach. Treatment allocation was based on clinical indication, surgeon judgment, and patient preference rather than randomization, which resulted in unequal group sizes (106 in the autologous fat grafting group and 98 in the alloplastic reconstruction group). A prior sample size estimation using an expected effect size of 0.35, 80% power, and 5% significance level suggested a minimum of 180 participants; therefore, the final cohort of 204 patients provided sufficient statistical power for comparative analysis. The study’s exploratory and real-world design, however, limited strict randomization or stratified recruitment. Nonetheless, the achieved sample size aligns with recent reconstructive and urogenital surgery series, such as Lai et al. (n = 20) and Laso-García et al. (n = 75) [13,14], supporting its adequacy for descriptive and comparative interpretation. This methodological consideration has been transparently acknowledged in the discussion section.

Data collection

All of the centers utilized the same technique for gathering data. Documentation was kept on postoperative problems (such as infection, extrusion, and fibrosis), surgical details, trauma characteristics, and demographics. A blinded panel used a validated aesthetic rating system to assess cosmetic results at 6 and 12 months after surgery. A validated 5-point Likert scale was used to measure patient-reported satisfaction at the same intervals. Structured interviews, surgery records, and patient files were used to gather data prospectively. Outcome assessors and the aesthetic rating panel were blinded to the type of procedure performed to minimize observer bias.

Surgical procedures and techniques

All surgical procedures were conducted in standardized operating conditions across participating centers, adhering to aseptic protocols and under either regional or general anesthesia based on patient tolerance and defect characteristics. Each procedure (Table 1) was performed by a multidisciplinary surgical team, consisting of one consultant plastic surgeon and one consultant urologist, supported by residents. In total, six consultants (three from plastic surgery and three from urology) participated, ensuring consistency and procedural reproducibility across all cases.

**Table 1:** Stepwise comparison of operative stages in autologous fat grafting versus alloplastic reconstruction procedures.

Step	Autologous Fat Grafting Group	Alloplastic Reconstruction Group
Preoperative Assessment	Defect dimensions, tissue elasticity, and donor site viability were assessed using ultrasound and manual examination.	Assessment of defect site, suitability for implant placement, and selection of implant or mesh type (silicone or polypropylene).
Anesthesia and Preparation	Regional or general anesthesia administered; surgical site sterilized and draped.	Similar anesthesia and preparation protocol applied.
Harvesting Phase	Fat harvested from lower abdomen or medial thigh using low-pressure suction with 3-mm cannula.	Implant or mesh prepared, sterilized, and pre-shaped to match defect contour.
Processing/Preparation	Harvested fat centrifuged at 3000 rpm for 3 minutes to separate adipose fraction.	Area of defect debrided, fibrotic or nonviable tissue removed.
Graft/Implant Insertion	Purified fat injected in micro-aliquots (35–45 mL) using multilayer, fan-shaped technique to ensure uniform distribution.	Silicone implant or polypropylene mesh inserted through 2–3 cm incision and secured with non-absorbable sutures (tension-free fixation).
Hemostasis and Drainage	Minimal electrocautery used; compression applied to reduce seroma formation.	Closed-suction drain placed in large dissection cases to prevent hematoma.
Wound Closure	Layered closure using absorbable sutures; sterile dressing applied.	Layered closure with absorbable sutures; sterile dressing and compression applied.
Postoperative Care	Antibiotic prophylaxis and analgesics given; early mobilization encouraged.	Similar antibiotic protocol; drain removed after 48–72 hours if output <30 mL.
Follow-Up	Evaluations at 1 week, 1 month, 6 months, and 12 months.	Same schedule followed; assessed for infection, fibrosis, or extrusion.

Follow-up and evaluation

Postoperative outcomes were assessed at standardized intervals using both clinical and imaging evaluations. Parameters recorded included graft/implant integration, local complications (infection, fibrosis, extrusion, necrosis), and aesthetic or functional satisfaction based on predefined scoring criteria. All complications were managed according to institutional protocols, and any revision procedures were documented.

Statistical analysis

Data analysis was conducted using IBM Corp. Released 2020. IBM SPSS Statistics for Windows, Version 26. Armonk, NY: IBM Corp. Baseline characteristics and results were described using descriptive statistics. For continuous data, the independent t-test was used, and for categorical variables, the chi-square test. P-values less than 0.05 were regarded as statistically significant. To account for confounding factors such as age, gender, the severity of the trauma, and the treatment location, multivariable regression was used.

Ethical approval

Ethical approval was obtained from the Institutional Review Boards (IRBs) of Shifa International Hospitals, Islamabad (641-728-2022). Written informed consent was obtained from all participants. Patient confidentiality was maintained in compliance with ethical research standards.

## Results

Table 2 shows that 106 of the 204 patients had autologous fat grafting and 98 had alloplastic repair. The fat grafting group's mean age was 36.4 ± 10.2 years, whereas the alloplastic group's was 38.1 ± 11.4 years (p = 0.218). Males made up 66.33% (n = 65) in the alloplastic group and 67.92% (n = 72) in the fat grafting group, indicating a comparable gender distribution (p = 0.811). 32.08% (n = 34) and 33.67% (n = 33) of the population were female. There was no significant difference in the mechanism of injury: penetrating injuries occurred in 27.36% (n = 29) and 25.51% (n = 25); iatrogenic injuries occurred in 33.96% (n = 36) and 37.76% (n = 37); blunt trauma affected 38.68% (n = 41) in the fat grafting group and 36.73% (n = 36) in the alloplastic group (all p > 0.5). Additionally, the mean duration between injury and operation was statistically similar (12.7 ± 5.4 vs. 13.1 ± 6.0 days, p = 0.563).

**Table 2:** Demographic and clinical characteristics of patients (N = 204). Baseline demographic and clinical variables were compared between autologous fat grafting and alloplastic material groups. An independent t-test used for continuous variables, and a chi-square test for categorical variables. No statistically significant differences were observed.

Category	Characteristic	Autologous Fat Grafting (n = 106)	Alloplastic Material (n = 98)	p-value
Demographics	Mean Age (years ± SD)	36.4 ± 10.2	38.1 ± 11.4	0.218
	Male, n (%)	72 (67.92%)	65 (66.33%)	0.811
	Female, n (%)	34 (32.08%)	33 (33.67%)	0.811
Injury Characteristics	Mechanism of Injury: Blunt, n (%)	41 (38.68%)	36 (36.73%)	0.771
	Mechanism of Injury: Penetrating, n (%)	29 (27.36%)	25 (25.51%)	0.774
	Mechanism of Injury: Iatrogenic, n (%)	36 (33.96%)	37 (37.76%)	0.575
Clinical Course	Time from Injury to Surgery (days)	12.7 ± 5.4	13.1 ± 6.0	0.563

The average fat volume injection for the fat grafting group was 38.6 ± 12.4 mL (Table 3). Of the patients in the alloplastic group, 47 (47.96%) had silicone implants and 51 (52.04%) received mesh implants. The alloplastic group's surgical time was 103.7 ± 20.9 minutes, whereas the fat grafting group's was 92.4 ± 18.1 minutes (p = 0.002). Similarly, the fat grafting group's average hospital stay was considerably shorter (1.8 ± 0.6 days) than the alloplastic group's (2.4 ± 1.1 days) (p = 0.001).

**Table 3:** Surgical parameters. Fat grafting required significantly shorter operative time and hospital stay compared to alloplastic repair. *p < 0.05 indicates statistical significance (independent t-test).

Parameter	Autologous Fat Grafting (n = 106)	Alloplastic Material (n = 98)	p-value
Average Fat Volume Injected (mL)	38.6 ± 12.4	N/A	—
Implant Type – Mesh, n (%)	N/A	51 (52.04%)	—
Implant Type – Silicone, n (%)	N/A	47 (47.96%)	—
Surgical Time (minutes ± SD)	92.4 ± 18.1	103.7 ± 20.9	0.002*
Hospital Stay (days ± SD)	1.8 ± 0.6	2.4 ± 1.1	0.001*

The fat grafting group had fewer postoperative problems (Table 4). Compared to 14 patients (14.29%) in the alloplastic group, five patients (4.72%) had infection (p = 0.018). Only in the alloplastic group (nine patients, 9.18%, p = 0.003) was graft/implant extrusion seen. Twelve patients (12.24%) in the alloplastic group and four patients (3.77%) in the fat grafting group developed fibrosis (p = 0.021). Seven (6.60%) and 11 (11.22%) patients had seromas, respectively (p = 0.216), and two (1.89%) and six (6.12%) patients needed reoperations, respectively (p = 0.142).

**Table 4:** Postoperative complications. Fat grafting was associated with significantly lower rates of infection, extrusion, and fibrosis compared to alloplastic repair. *p < 0.05 indicates statistical significance (chi-square test).

Complication	Autologous Fat Grafting (n = 106)	Alloplastic Material (n = 98)	p-value
Infection, n (%)	5 (4.72%)	14 (14.29%)	0.018*
Graft/Implant Extrusion, n (%)	0 (0.00%)	9 (9.18%)	0.003*
Fibrosis, n (%)	4 (3.77%)	12 (12.24%)	0.021*
Seroma Formation, n (%)	7 (6.60%)	11 (11.22%)	0.216
Reoperation Required, n (%)	2 (1.89%)	6 (6.12%)	0.142

Table 5 shows that 24 patients (24.49%) in the alloplastic group and 49 patients (46.23%) in the fat grafting group had an outstanding aesthetic assessment (5/5) at six months (p = 0.001). These figures increased to 31 (31.63%) and 61 (57.55%) at 12 months (p = 0.001). At six months (30.19% vs. 35.71%, p = 0.393) and 12 months (27.36% vs. 33.67%, p = 0.315), the percentage classified as "good" (scoring 4) was comparable. 22 (20.75%) vs. 30 (30.61%) at six months (p = 0.112) and 14 (13.21%) vs. 27 (27.55%) at 12 months (p = 0.011) indicated fair results (scoring 3). At six months, the fat grafting group had substantially fewer poor outcomes (scores 1-2) (2.83% vs. 9.18%, p = 0.049), with a nonsignificant trend at twelve months (1.89% vs. 7.14%, p = 0.090).

**Table 5:** Aesthetic outcome at 6 and 12 months (blinded panel assessment). A blinded panel assessment of aesthetic results demonstrated superior outcomes for fat grafting, particularly in excellent ratings and reduced poor results. *p < 0.05 indicates statistical significance (chi-square test).

Aesthetic Outcome (Score 1–5)	Time Point	Autologous Fat Grafting (n = 106)	Alloplastic Material (n = 98)	p-value
Excellent (Score 5), n (%)	6 months	49 (46.23%)	24 (24.49%)	0.001*
	12 months	61 (57.55%)	31 (31.63%)	0.001*
Good (Score 4), n (%)	6 months	32 (30.19%)	35 (35.71%)	0.393
	12 months	29 (27.36%)	33 (33.67%)	0.315
Fair (Score 3), n (%)	6 months	22 (20.75%)	30 (30.61%)	0.112
	12 months	14 (13.21%)	27 (27.55%)	0.011*
Poor (Score 1–2), n (%)	6 months	3 (2.83%)	9 (9.18%)	0.049*
	12 months	2 (1.89%)	7 (7.14%)	0.090

The fat grafting group had greater patient satisfaction (Table 6). In the alloplastic group, 45 patients (42.45%) reported being extremely pleased (scoring 5) at six months, compared to 22 (22.45%) (p = 0.001), and this number rose to 58 (54.72%) vs. 29 (29.59%) after 12 months (p = 0.001). At six months (33.02% vs. 34.69%, p = 0.812) and 12 months (29.25% vs. 35.71%, p = 0.322), there was no discernible change in the "satisfied" (score 4) group. 18 (16.98%) and 26 (26.53%) patients at six months (p = 0.108) and 13 (12.26%) and 21 (21.43%) at 12 months (p = 0.098) had neutral reactions (scoring 3). At both time intervals, the alloplastic group's dissatisfaction (score 2) was somewhat greater (6 months: 11.22% vs. 5.66%; 12 months: 9.18% vs. 3.77%), but this difference was not statistically significant. Interestingly, after 12 months, four (4.08%) patients in the alloplastic group had high levels of dissatisfaction (scoring 1), but none in the fat grafting group did (p = 0.045).

**Table 6:** Patient-reported satisfaction at six and 12 months (5-point Likert scale). Patient-reported satisfaction favored fat grafting, with significantly more “very satisfied” responses and fewer “very dissatisfied” responses compared to alloplastic repair. *p < 0.05 indicates statistical significance (chi-square test).

Satisfaction Score	Time Point	Autologous Fat Grafting (n = 106)	Alloplastic Material (n = 98)	p-value
Very Satisfied (Score 5)	6 months	45 (42.45%)	22 (22.45%)	0.001*
	12 months	58 (54.72%)	29 (29.59%)	0.001*
Satisfied (Score 4)	6 months	35 (33.02%)	34 (34.69%)	0.812
	12 months	31 (29.25%)	35 (35.71%)	0.322
Neutral (Score 3)	6 months	18 (16.98%)	26 (26.53%)	0.108
	12 months	13 (12.26%)	21 (21.43%)	0.098
Dissatisfied (Score 2)	6 months	6 (5.66%)	11 (11.22%)	0.104
	12 months	4 (3.77%)	9 (9.18%)	0.128
Very Dissatisfied (Score 1)	6 months	2 (1.89%)	5 (5.10%)	0.421
	12 months	0 (0.00%)	4 (4.08%)	0.045*

## Discussion

Alloplastic materials and autologous fat grafting were examined in this observational research for soft tissue restoration after urogenital injuries. According to our research, autologous fat grafting produces better results in terms of patient satisfaction, cosmetic appearance, and complications. These findings are consistent with the increasing amount of data that fat grafting is a safe and biocompatible substitute for traditional reconstructive surgery.

The autologous fat grafting group saw considerably fewer postoperative complications. Just 4.72% of fat transplant recipients had infection, while the alloplastic group had 14.29% (p = 0.018). Additionally, 9.18% of alloplastic patients had implant extrusion, but the fat grafting group had no extrusion recorded (p = 0.003). These results are in line with earlier studies conducted in the perineal area, which showed that autologous fat grafting was safe for urogenital soft tissue repair due to its low rates of infection and extrusion [15].

Additionally, fat grafting was preferred for aesthetic results as assessed by blinded scoring. Compared to 24.49% of patients in the alloplastic group, 46.23% of patients in the fat grafting group had an excellent aesthetic score (5/5) after six months (p = 0.001). At 12 months, this difference remained (57.55% vs. 31.63%, p = 0.001). Comparable investigations found that autologous procedures were more satisfactory for reconstructing the penis and perineum, and they attributed this to improved tissue pliability and integration [16,17]. According to our research, fibrosis was considerably less common in the fat grafting group (3.77% vs. 12.24%, p = 0.021). This is in line with earlier findings, which indicate that fat grafting enhances tissue pliability and decreases fibrotic remodeling in radiation-induced soft tissue injury by fostering the development of a regenerative microenvironment [18].

In the autologous group, patient satisfaction was much greater. A total of 54.72% of fat grafting patients reported being "very satisfied" with their results after 12 months, compared to 29.59% in the alloplastic group (p = 0.001). Interestingly, 4.08% of alloplastic recipients reported feeling "very dissatisfied," compared to 0.045% for fat grafting patients. These results support earlier research that highlighted the physical and psychological advantages of autologous fat in intimate area repair [19].

Additionally, our results demonstrated benefits in terms of recovery and operating efficiency: the fat grafting group saw a substantial reduction in both surgery time and hospital stay (92.4 ± 18.1 minutes vs. 103.7 ± 20.9, p = 0.002; 1.8 ± 0.6 days vs. 2.4 ± 1.1, p = 0.001). These useful advantages increase the allure of fat grafting even more, particularly in environments with limited resources.

Strengths and limitations

This study's multicenter design, which improves the results' generalizability across various clinical settings, is one of its main strengths. Real-time evaluation of surgical results and patient satisfaction was made possible by the prospective observational technique, which offered a thorough analysis of both objective and subjective factors. A 12-month follow-up and the use of standardized ratings for aesthetic and satisfaction results strengthen the comparison study. The study also has certain limitations. Despite being sufficient, the sample size can still restrict subgroup analysis, particularly for uncommon problems. The absence of randomization raises the possibility of selection bias, and variations in surgeon specialization throughout centers could have affected the outcomes. To validate these results, lengthier follow-up periods in future randomized controlled studies are necessary.

## Conclusions

This research shows that when it comes to the treatment of post-traumatic urogenital soft tissue abnormalities, autologous fat grafting provides noticeably superior reconstructive results than alloplastic materials. In addition to shorter recovery periods and hospital stays, patients who received fat grafts reported better cosmetic outcomes, fewer problems, and higher satisfaction ratings. These results provide credence to the increasing use of autologous methods in intimate-area repair, especially in areas that are anatomically and psychologically delicate.
